# An evaluation of UK foundation trainee doctors’ learning behaviours in a technology-enhanced learning environment

**DOI:** 10.1186/s12909-016-0651-z

**Published:** 2016-05-03

**Authors:** Hannah L. Brooks, Sarah K. Pontefract, James Hodson, Nicholas Blackwell, Elizabeth Hughes, John F. Marriott, Jamie J. Coleman

**Affiliations:** College of Medical and Dental Sciences, University of Birmingham, Birmingham, B15 2TT UK; University Hospitals Birmingham NHS Foundation Trust, Edgbaston, Birmingham, B15 2TH UK; OCB Media Ltd, The Crescent, 27 King Street, Leicester, LE1 6RX UK; Health Education England’s West Midlands team, St Chads Court, 213 Hagley Road, Edgbaston, Birmingham, B16 9RG UK

**Keywords:** Foundation trainees, Doctors, Prescribing, Technology enhanced learning, eLearning, Learning behaviours

## Abstract

**Background:**

Technology-Enhanced Learning (TEL) can be used to educate Foundation Programme trainee (F1 and F2) doctors. Despite the advantages of TEL, learning behaviours may be exhibited that are not desired by system developers or educators. The aim of this evaluation was to investigate how learner behaviours (e.g. time spent on task) were affected by temporal (e.g. time of year), module (e.g. word count), and individual (e.g. knowledge) factors for 16 mandatory TEL modules related to prescribing and therapeutics.

**Methods:**

Data were extracted from the SCRIPT e-Learning platform for first year Foundation trainee (F1) doctors in the Health Education England’s West Midland region from 1^st^ August 2013 to 5^th^ August 2014. Generalised Estimating Equation models were used to examine the relationship between time taken to complete modules, date modules were completed, pre- and post-test scores, and module factors.

**Results:**

Over the time period examined, 688 F1 doctors interacted with the 16 compulsory modules 10,255 times. The geometric mean time taken to complete a module was 28.9 min (95 % Confidence Interval: 28.4–29.5) and 1,075 (10.5 %) modules were completed in less than 10 min. In February and June (prior to F1 progression reviews) peaks occurred in the number of modules completed and troughs in the time taken. Most modules were completed, and the greatest amount of time was spent on the learning on a Sunday. More time was taken by those doctors with greater pre-test scores and those with larger improvements in test scores.

**Conclusions:**

Foundation trainees are exhibiting unintended learning behaviours in this TEL environment, which may be attributed to several factors. These findings can help guide future developments of this TEL programme and the integration of other TEL programmes into curricula by raising awareness of potential behavioural issues that may arise.

**Electronic supplementary material:**

The online version of this article (doi:10.1186/s12909-016-0651-z) contains supplementary material, which is available to authorized users.

## Background

Foundation trainee doctors (F1 and F2; those in their first two years after qualifying with a medical degree from university) in the UK are currently allocated protected learning time during each working week [1], of which some activities can be mandated. Given that prescribing is one of the activities most frequently undertaken by Foundation trainees, and that trainees often feel underprepared to prescribe following their undergraduate education [2–6], it has been recommended that some time should be dedicated to prescribing education [7].

Keogh et al. [8] found that the most frequent educational activity voluntarily undertaken by Foundation trainees was via Technology-Enhanced Learning (TEL) packages and it likely that the use of technology in medical education will continue to increase [9]. There are many advantages associated with TEL, including ease of access and the permitted flexibility in the time and place of learning, [10–13]. Such ‘just-in-time’ learning allows learners to obtain the specific information needed to achieve a goal, as and when they require it [14–16]. This may be beneficial for busy trainees, where time is often a limiting factor in undertaking formal education [8, 17]. Indeed, TEL has been widely advocated for the education of medical professionals in a variety of settings and circumstances [18–21].

Despite the advantages of TEL, such flexibility may allow for busy Foundation trainees to exhibit inappropriate learning behaviours within the learning environment. Learners may not complete the TEL as recommended (e.g. completing content only directly prior to an assessment) or use system workarounds (e.g. skipping over content), thereby facilitating superficial as opposed to deep learning, if any learning at all. It is important to determine specifically what may occur in order to aid with future developments of TEL programmes, which has not been discussed in previous research. In Temporal Motivation Theory [22] (TMT), it is suggested that motivation to complete a task is affected by the individual’s perception of their probability of achieving success; the salience of the reward or punishment associated with completing or not completing the task, and the salience of a potential reward or punishment in relation to its temporal proximity. Therefore, Foundation trainees may only be motivated to complete TEL activities if they will be strongly rewarded (e.g. gaining additional credits) or punished (e.g. failing a course) as a result of an imminent task (e.g. an exam they must take tomorrow). By allowing little time to complete work, other learning behaviours (e.g. time spent on the task) may be compromised. TMT may serve to account for some of the learning behaviours that may be observed in a mandated ‘just-in-time’ learning context.

It is important to evaluate learning behaviours in order to identify those that may be suboptimal. This can help to improve education, since a lack of engagement may be associated with poor performance. This may be especially pertinent in unmonitored TEL environments, to ensure that the appropriate learning is undertaken and to inform future instructional design. Owing to *ad hoc* monitoring of users’ interactions with the system, we identified a need to evaluate exactly this. As such, we investigated how Foundation trainee doctors’ learning behaviours (e.g. time spent on task; frequency of task completion) were affected by temporal (e.g. time of year), module (e.g. the quantity of learning material), and individual (e.g. knowledge) factors for a range of mandatory TEL modules relating to prescribing and therapeutics.

## Method

### Background

Online learning for Foundation trainee doctors is commonly used across the UK to help prepare them for practice in various areas of healthcare practice. SCRIPT is an e-learning resource used by Foundation trainee doctors across various regions in England (www.safeprescriber.org). Within SCRIPT, there are currently 45 available modules, designed to educate Foundation trainee doctors about prescribing in a variety of patient populations and situations. Modules include topics such as Prescription Documentation, Prescribing in Renal Dysfunction, and Adverse Drug Reactions. The modules were developed by a multidisciplinary team of healthcare professionals and are updated on a regular basis. Each module comprises a set of multiple choice questions (‘pre-test’), the main body of learning content, a repeat set of identical questions presented in a random order (‘post-test’), and some suggested further reading. Following completion of the post-test, a certificate of completion can be viewed and downloaded by the user for their online learning portfolio (ePortfolio), which they are required to complete during the Foundation training programme.

In August 2011, it became mandatory for Foundation Year 1 (F1) doctors in the West Midlands to complete 16 specified modules within this first year of training. No other instruction about the order of module completion or time frame for completing modules was specified. Bi-annual progression review meetings are held between Foundation trainees and their clinical tutor in early March and early June, for which the trainee must have prepared specific items in their ePortfolio for submission and evaluation.

Through the SCRIPT e-Learning platform, it is possible to monitor individual user activity. It is possible to identify the date on which individual modules are completed (pre-test and post-test completion are monitored independently), the length of time spent engaging with the learning content, and both pre-test and post-test scores. Clinical tutors (for their tutees only) and administrators (for all users) are able to access this data.

### Data capture

For the purpose of the current evaluation, data from 1^st^ August 2013 to 5^th^ August 2014 was extracted for each F1 doctor in the West Midlands, and anonymised by the system developers. Data included information on the date of module completion, the time taken to complete the module, and the pre- and post-test scores for each of the 16 mandatory SCRIPT modules. Data were also collated on the word count, number of images, and number of web links available (excluding further reading) for each module. The module titles are displayed in Table 1. All specified modules are considered core, and aligned to the standards expected during the F1 year of training. Ethical approval was gained from the University of Birmingham [reference number: ERN_14-0746SB]. Trainees consent to the Privacy Policy upon registration, which states that Health Education England may use the data to get a sense of how people interact with the site.

**Table 1:** Foundation Year 1 (F1) Mandatory SCRIPT Module Titles

Module Title	
Adverse Drug Reactions	Prescribing in Renal Dysfunction
Anticoagulation	Prescribing in Older Adults
Dosing and Calculation	Pain Management
Drug Allergy and Anaphylaxis	Parenteral Poisons
Drug Interactions	Prescribing in Infection
Fluids	Rational Drug Choice
Medication Errors	Taking a Safe and Effective Drug History
Prescription Documentation	Toxic Tablets

It is important to note that all medical graduates must achieve a specified standard in order to commence the Foundation training programme. Although it is expected that all users would have a similar level of competence prior to completing the modules, there may be some between-trainee variability. Our data capture therefore examined individual progression from pre- to post-test scores.

### Analysis

Owing to the level of skew in the distribution of the times taken to complete modules, the variable was log_10_ transformed prior to analysis. Since each doctor took multiple modules, it was not reasonable to assume that all instances in the data were independent. For this reason, the data were analysed using Generalised Estimating Equations (GEE), with an exchangeable correlation structure, in order to account for the within-doctor correlations in the times taken to complete modules. A range of temporal- and module-related factors were included as independent variables in this model, to test for associations with module completion time. The coefficients from this model were then anti-logged, and converted into estimates of the geometric mean module completion times, after accounting for the effects of the other factors in the model as well as the within-doctor correlations.

Factors relating to module content were then collated, and compared to the estimated averages from the GEE model using Spearman’s correlation coefficients. Variables with significant correlations were then entered into a linear regression model.

All analyses were performed using IBM SPSS 19 (IBM Corp. Armonk, NY), with *p* < 0.05 deemed to be indicative of statistical significance.

## Results

### Distribution of modules over time

**Fig. 1:**
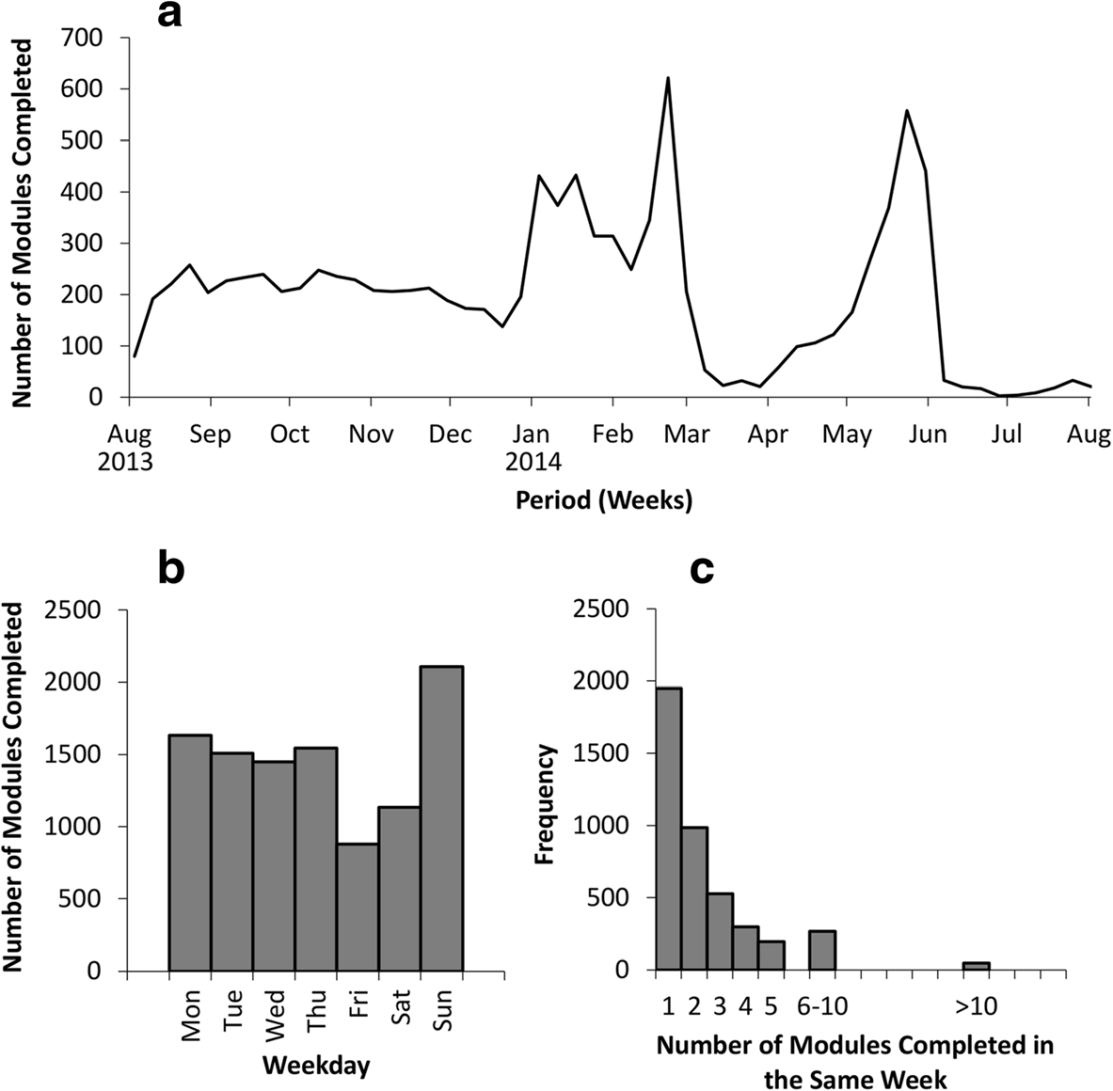
Relationship between module completion and time of year (1a); day of week (1b); and the clustering of modules within the same calendar week (1c)

Over the evaluation period, 688 F1 doctors interacted with the modules 10,255 times. Figure 1a shows how these modules were distributed over time. The numbers of modules completed per week appeared to be relatively constant, at around 200 modules per week from the start of the study in August 2013 to the end of the year. At the beginning of 2014, there was a sharp increase, peaking in late February 2014, with 622 modules completed in a week. Subsequently, the numbers of modules completed fell to the lowest levels throughout March, with a minimum of 21 completed in a week, before rising again to another peak of 558 per week by the end of May. Of the 688 trainees, 583 completed all 16 mandatory modules within their F1 year. Of these, 53.2 % had completed all of their modules by the beginning of May, which increased to 97.6 % by the end of May. The trends in the frequency of module completion were similar in 14 of the 16 mandatory modules; two modules differed (Additional file 1: Figure S1). These modules, *Taking a Safe and Effective Drug History* and *Prescription Documentation*, were most frequently completed within the first three months of the Foundation year (August to October).

Figure 1b shows how the modules taken were distributed over the days of the week. The numbers were similar for Monday through Thursday, at around 1,500 modules on each day. Friday and Saturday were the days where least modules were taken, with totals of 878 and 1,133 respectively. However, Sunday was found to be the day that F1 doctors most commonly completed modules, with a total of 2,107.

Figure 1c shows the clustering of modules within the same calendar week. For each module completed, the total number of other modules completed in the same week was calculated. There were a number of instances where a large number of modules were completed in a batch during the same week. There were 49 instances of more than 10 modules being completed in the same calendar week, of which nine represented doctors completing all 16 of their modules within the same week. Of these, one trainee completed their modules in August 2013, one in October 2013, two in January 2014, one in February 2014 and four in May 2014.

### Time taken to complete modules

The log-transformed times to complete modules closely followed a normal distribution, however there were more modules than expected that were completed quickly. The geometric mean time taken to complete modules was 28.9 min (95 % Confidence Interval (CI): 28.4–29.5), with 1,075 (10.5 %) modules being completed in under 10 min. The shortest time to complete a module was 38 s. These short times also resulted in an asymmetrical distribution, which contravened the assumptions of parametric analytical techniques. For this reason, modules that were completed in less than five minutes (*N* = 497, 4.8 %) were excluded from the analysis of module completion times. After excluding these cases, the geometric mean completion time of the remaining modules increased to 32.9 min (95 % CI: 32.5–33.4).

The GEE model demonstrated a moderate level of within-doctor correlation (coefficient = 0.484). After accounting for this, all of the factors considered (module; pre-module score; change in score from pre-test to post-test; weekday; number of modules completed per week; time period) were found to be significant at *p* < 0.001.

**Fig. 2:**
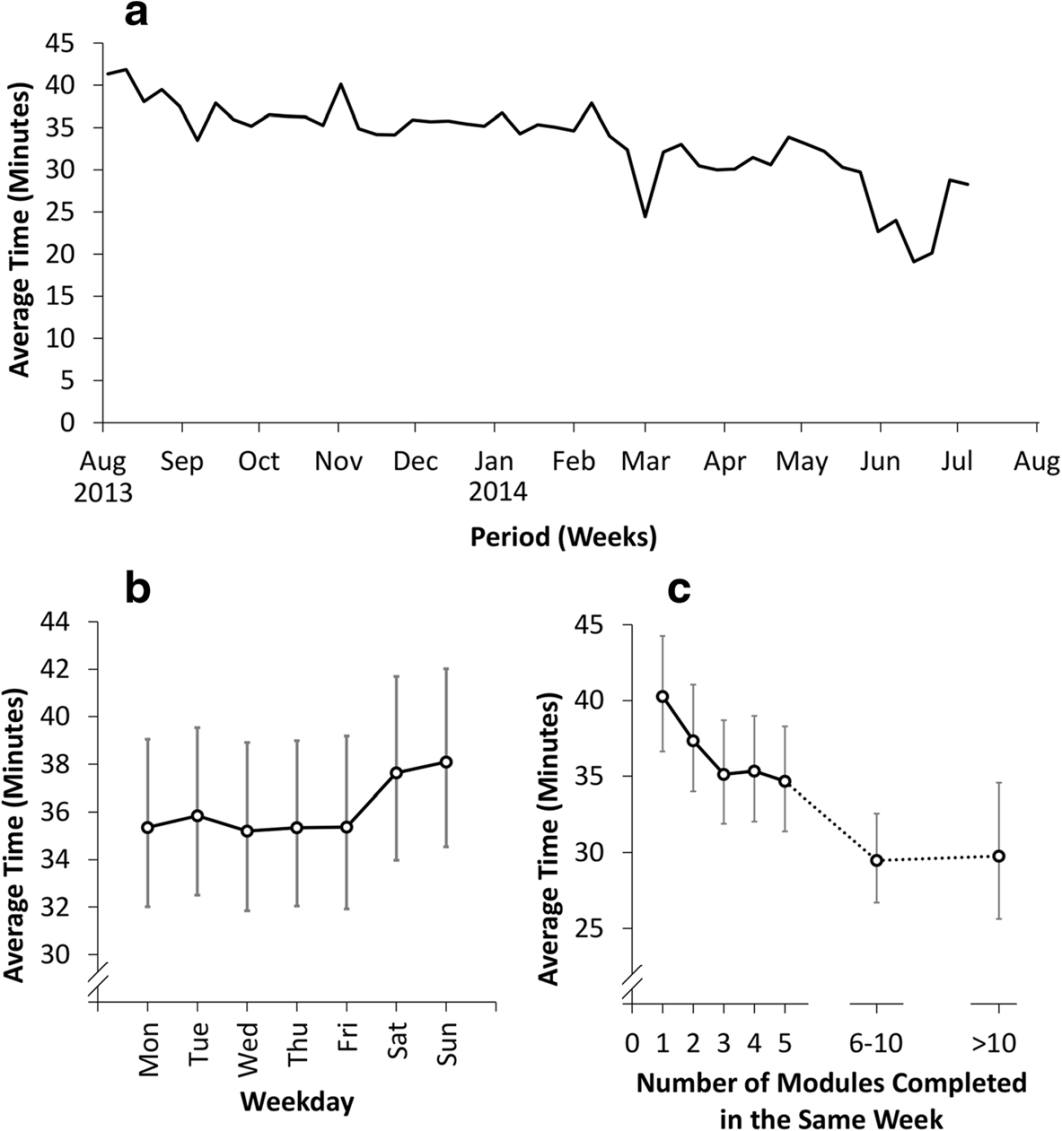
Relationships between month of the year (2a), day of the week (2b), and number of modules completed in the same week (2c) and average module completion times. *Footnote: Plotted values are geometric mean times based on the parameter estimates from the GEE model, after accounting for the module name, pre-module score, change in score, weekday, number of modules completed in the same week, and the within-doctor correlation. Whiskers represent 95 % confidence intervals*

Figure 2 illustrates the relationship between temporal factors and the times taken to complete modules. Figure 2a shows a gradual reduction in the time taken to complete modules over the course of the study, from a geometric mean of 41.3 min (95 % CI: 35.4–48.2) in the first week, to 33.9 min (95 % CI: 29.4–39.0) by the end of April 2014. After this, there is a clear trough, with the geometric mean time falling to 22.7 min (95 % CI: 20.0–25.7) by the end of the following month. Another trough is observed in early March 2014. These two troughs coincide with the peaks in the numbers of modules being taken that were identified previously. This is consistent with the finding that the greater number of modules completed in the same week, the less time was spent on each module (Fig. 2c), with the geometric mean completion time taken falling from 40.3 min (95 % CI: 36.7–44.3) where only one module is completed in a week, to 29.8 min (95 % CI: 25.6–34.6) where more than ten are completed.

Figure 2b illustrates that, whilst the average time taken to complete modules was relatively constant across each week, there was a small increase over the weekend, with geometric means of 35 min for weekdays, 37.6 min on Saturday and 38.1 min on Sunday.

**Fig. 3:**
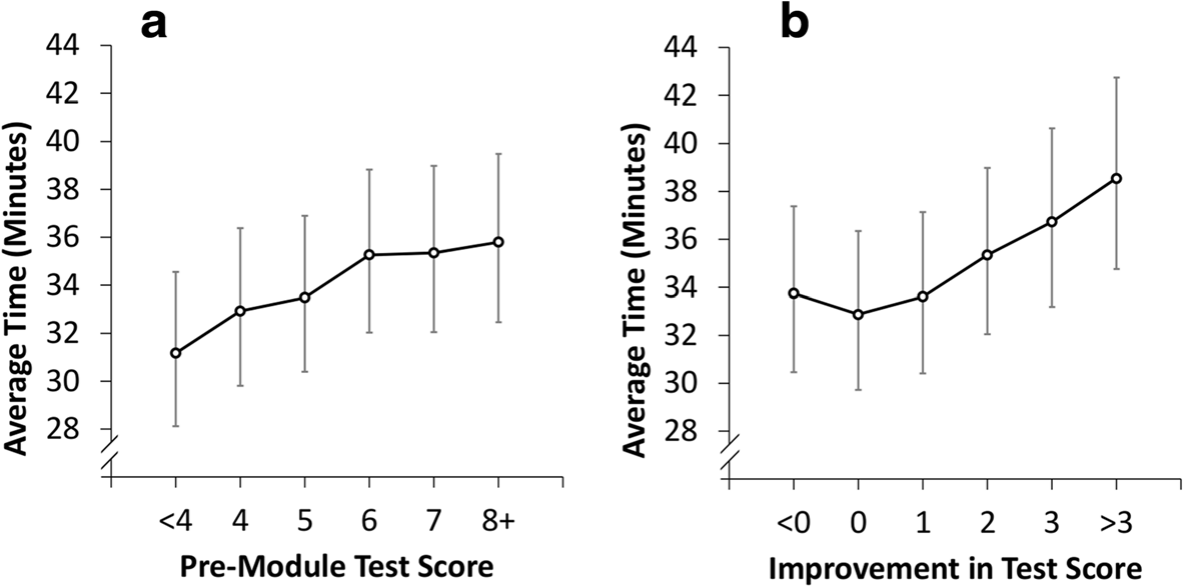
Relationships between pre-module test scores (3a) and improvement in test scores (3b) and average module completion times. *Footnote: Plotted values are geometric mean times based on the parameter estimates from the GEE model, after accounting for the module name, pre-module score, change in score, weekday, number of modules completed in the same week, and the within-doctor correlation. Whiskers represent 95 % confidence intervals*

As shown in Fig. 3a and 3b, respectively, more time was spent completing modules by those users who had a greater pre-test score and by those who showed greater improvement from pre-test to post-test scores.

**Fig. 4:**
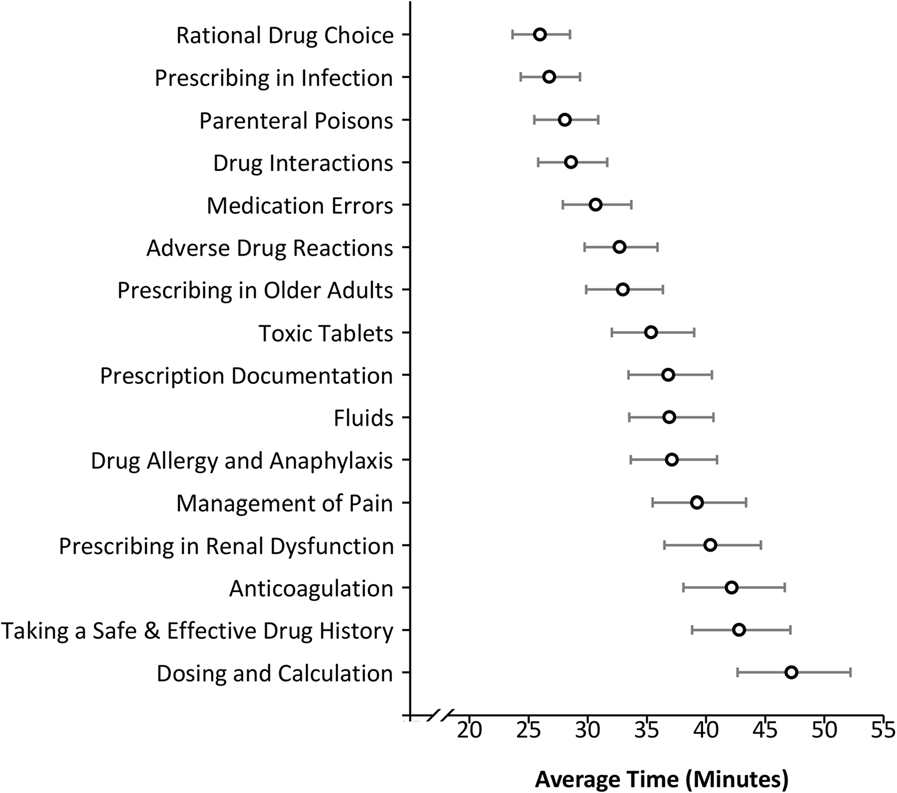
Average completion times by module. *Footnote: Plotted values are geometric mean times based on the parameter estimates from the GEE model, after accounting for the module name, pre-module score, change in score, weekday, number of modules completed in the same week, and the within-doctor correlation. Whiskers represent 95 % confidence intervals*

Figure 4 illustrates how the completion times taken differed by module. As can be seen, the least time was spent on ‘Rational Drug Choice’ (geometric mean: 26.0 min, 95 % CI: 23.6–28.5), and the most time was spent on ‘Dosing and Calculation’, which took almost twice as long, on average (geometric mean: 47.2 min, 95 % CI: 42.7–52.2).

The correlation coefficients between the content-related factors, and the geometric mean times for each module from the GEE model were also examined. The only factor found to be significant was the module word count, with a Spearman’s correlation coefficient of 0.756 (*p* < 0.001). A linear regression model found that the average time taken to complete a module increased by 2.8 min per 1,000 words (95 % CI: 1.4–4.3, *p* = 0.001).

## Discussion

Using data from a content management system, we investigated Foundation trainee doctors’ learning behaviours when completing TEL modules for prescribing education. A number of results indicate that suboptimal learning behaviours were being exhibited by trainees, demonstrated by changes to the frequency of module completion and the time spent on learning over time, across modules, and individuals.

Regarding the frequency of module completion, peaks existed in February and June, which appeared to occur around the time of trainees’ biannual progression reviews with their clinical tutor. This suggests a large number of modules were being completed in response to an upcoming or recently completed review. As Trusts’ review periods may differ slightly depending on the number of trainees in the hospital and availability of both trainees and Clinical Tutors, this may also account for a prolonged peak in the increased frequency of completion. Furthermore, in the month prior to the second review period there was a rapid increase in the number of trainees who had completed all 16 of their mandatory modules. These findings support Temporal Motivation Theory [22], as the review would act as a salient and temporally proximal event leading to reward or punishment, meaning that trainees would experience increasing motivation to complete the modules leading up to the review. The reviews may also provide some explanation as to the behaviour of the nine trainees who completed all 16 mandatory modules within one week – of these cases, two trainees completed their modules in January, one completed them in February, and four completed them in May, which would have been just prior to their review meeting. This finding highlights the drawbacks of the flexibility of TEL, as it is not possible to ensure that modules are completed as they were designed and intended, or distributed throughout the year appropriately.

Of the 688 F1 trainees, 105 did not complete all of the mandatory modules. The modules must be completed before progressing into the Foundation Year 2 (F2) training; therefore it is unlikely that this was due to trainees simply not completing modules without good reason. For example, some trainees may have completed some of the modules at their undergraduate institution and therefore were exempt from completing these again in their F1 year. Furthermore, some trainees may have taken extended leave or left the Foundation training programme and therefore not completed all of the mandatory modules within the study period. It would be interesting to investigate these cases in more detail.

Despite the findings that many modules may have been completed in response to tutor reviews, some modules were more likely to be completed early in the year (e.g. *Prescription Documentation*, *Taking a Safe and Effective Drug History*). This may have been in response to trainees’ perceived educational needs, or based on individual Trusts’ requirements to complete these specific modules (which may be considered introductory) at the start of the year. Furthermore, modules are grouped together into seven sections (e.g. *Principles of Prescribing*; *Prescribing in Special Circumstances*) within the online programme. The two modules completed earlier in the year are located in the first section (*Principles of Prescribing*), which encompasses what may be considered the most basic material and is located at the top of the navigation pane. Therefore, they may have been completed earlier in the year based on their salience in the online learning environment, the relative ease of the content, or the necessity of the knowledge for trainees, which are factors we did not consider in our analyses.

It was also found that there was an increased frequency of module completion on Sundays compared to other days of the week, and that more time was spent on modules on Saturdays and Sundays. Trainees were utilising their free time during weekends, when they were presumably less busy, to dedicate time to TEL. This suggests a degree of intrinsic motivation for completing the learning, but also suggests that modules were not being completed during allocated study time during the working week.

Over the study period, the average time to complete a module declined. This may indicate that trainees are becoming more familiar with using the TEL programme, that they are becoming more confident and competent in their prescribing knowledge, and/or that the time and effort that they put in declines over the year. However, given that the average time for completing a module was 28.9 min and that 10.5 % of all modules were being completed in less than 10 min, it could be suggested that at least some of these cases can be attributed to a lack of time and effort rather than greater system or content expertise. The mean time is likely to be representative of the expected length of each module and significantly shorter times would seem to suggest a lack of engagement with the activities. The shortest time taken to complete a module (38 s) was surprising; there are no modules that are substantially short enough to warrant the interactions observed by some trainees. However it was found that it is physically possible to complete the modules in this time by rapidly navigating the learning system and avoiding interacting with optional module content. Clearly this does not represent doctors correctly using the educational tool. Furthermore, two troughs were evident in the average time taken to complete modules in late February and late May, which also correspond with the progression review dates. This provides evidence for a systematic decrease in the time taken to complete modules, which may be somewhat attributed to trainees rushing to complete numerous modules prior to their reviews. However, a greater word count was associated with more time being spent on the module, which provides some evidence that modules are in fact being utilised as intended.

Importantly, the results also highlighted that those doctors who spend little time completing one module tended to complete other modules quickly, and vice versa (within-doctor correlation from the GEE model of 0.484). Those trainees who spent less time completing modules had, on average, lower pre-test scores and showed smaller improvements in their knowledge from pre-test to post-test, compared to trainees who spent more time completing modules. The first of these findings may indicate that it is the more competent doctors who are more highly motivated to spend time on the TEL modules, or that those highly competent doctors also have better time management skills and are therefore able to spend more time working through each module. The second finding highlights that spending inadequate time completing the modules negates the purpose of the TEL programme, as prescribing knowledge (as measured by improvements on the multiple choice question test scores) is not improved to its full capacity. Those trainees spending more time completing modules are experiencing greater educational benefits, which may translate to the prescribing behaviours that are exhibited on the ward.

Based on these findings, it is possible to suggest that there is a need to regulate more closely when modules are completed throughout the year, so that modules are not being rapidly completed as a response to an upcoming review. Nonetheless, there is a need for the learning to remain flexible and based upon trainees’ needs at a particular time of year during the first year of the Foundation Programme, meaning it may be too challenging to mandate exactly when modules must be completed. Regarding the time taken to complete modules, there have already been some changes made to this system. For example, from June 2013, it became mandatory for users to view every page of a module, and users who take less than 10 min to complete a module are flagged on the e-learning platform, which their clinical tutor has the ability to view, Nonetheless, it is impossible to control how long a user actually spends on the learning, given that they are not required to physically attend a classroom-based session to complete the TEL. In the future, it may be possible to introduce a ‘code of conduct’ to make users aware of the expectations associated with SCRIPT, which may encourage Foundation trainees to utilise the TEL programme as intended.

### Implications for educators

The findings from this study highlight the need for educators to devise methods to encourage optimal behaviours during the implementation of any TEL programme. Careful consideration should be given to the integration of such learning into a curriculum and the recommendations given as to how and when the learning should be completed.

If the learning is mandatory but is completed during the learner’s free time, educators should consider providing instruction as to how long the learning should take and when it should be completed, in a specified order (if appropriate) over a specified time frame (e.g. per week or per year). Alternatively, time could be allocated for the learning to be completed. It is also important to regularly monitor individual learners’ progress within the learning environment (i.e. whether they are completing learning at an appropriate time point and engaging with the TEL environment for a suitable length of time), from which feedback can be provided to the learner. In line with Temporal Motivation Theory [22], regular deadlines and active monitoring of progress may help to motivate learners to complete the learning in a timely manner.

As a result of our findings, we have implemented clearer guidance for postgraduate centre managers and trainers about integrating TEL with other training opportunities. We have also provided Clinical Tutors and Foundation trainee doctors with clearer guidance on the use of the programme, and we have suggested that tutors regularly review completeness of the e-Learning modules and incorporate discussion of prescribing competence in their progress reviews, thus discouraging adverse temporal completion.

### Limitations and future research

In this study we only investigated mandatory TEL modules, and did not consider additional optional modules, which could provide additional or alternative explanations of learner behaviours. Second, it is only possible to hypothesise about Foundation trainees’ rationale for completing modules quickly. Furthermore, it is not possible to identify whether trainees are attending to the module content, even if they are technically engaged in the programme, or if they are completing the module alone. Therefore, the recorded time spent on a module can only be considered a surrogate measure of actual time spent engaged with the learning material. It is inevitable that work commitments will also influence trainee engagement with the programme. Whilst we make reference to temporal factors over the calendar period, due to the anonymity of trainees, we were unable to link specific doctors to rotations and therefore frequency of on calls. Lastly, learners’ engagement with TEL, particularly on screen activities and audio visual material, will vary according to their preferential learning styles. However, given the mandatory nature of the modules and lack of flexibility of the learning environment, it is difficult to take account of this variation.

In future research, to understand why trainees exhibit such learning behaviours, it would be interesting to further investigate whether there are any factors (e.g. personality, medical training background, clinical rotation and on-call commitment) shared among trainees who spend more or less time completing modules. In order to understand if there is a rationale for the patterns of behaviour observed in this study, it would also be useful to explore user attitudes towards the TEL programme, their interactions with the programme (e.g. completing modules with multiple or single interactions), and their motivation for its use. It may be important to gain an understanding of whether the mandatory nature of the learning had any effect on the observed trends in time taken, or the time of year that modules were completed.

Finally, it would be interesting to investigate whether the prescribing information provided to trainees in the TEL programme was retained and utilised in clinical practice, and if this had any effects on a larger scale (e.g. an overall reduction in medication errors within a hospital).

## Conclusion

These results lead to the suggestion that some Foundation trainees were exhibiting unintended learning behaviours in this TEL environment. Such behaviours may be attributed to several factors, including a lack of time during the working week, upcoming review meetings, prior knowledge, and system factors such as module length. The findings from this evaluation can be used to guide future developments of this TEL programme and the rules and regulations surrounding its use. This may help to ensure that Foundation trainees are undertaking appropriate learning in a timely manner, which will ultimately help to improve their knowledge of prescribing and subsequent prescribing behaviours. These findings will also hopefully raise awareness of potential behavioural issues that may be encountered when designing mandated TEL programmes for continued medical education.

### Ethics approval and consent to participate

Ethical approval was gained from the University of Birmingham [reference number: ERN_14-0746SB]. Upon registration, the users of the web-based programme consent to the *Privacy Policy*, which states that Health Education England may use the data to get a sense of how people interact with the site.

### Availability of data and materials

Upon registration, the users of the web-based programme consent to the *Privacy Policy*, which states that Health Education England may use the data to get a sense of how people interact with the site. The users have not consented to this data being publicly available for further research.

## Additional file

Additional file 1: Figure S1. Number of modules completed per week during Foundation Year 1 training (2013–14). (TIFF 376 kb)
