# Supplementary figures and images for: An evaluation of UK foundation trainee doctors’ learning behaviours in a technology-enhanced learning environment
Source: BMC Med Educ. 2016 May 3;16:133. doi: 10.1186/s12909-016-0651-z (PMC4855751; doi:10.1186/s12909-016-0651-z)

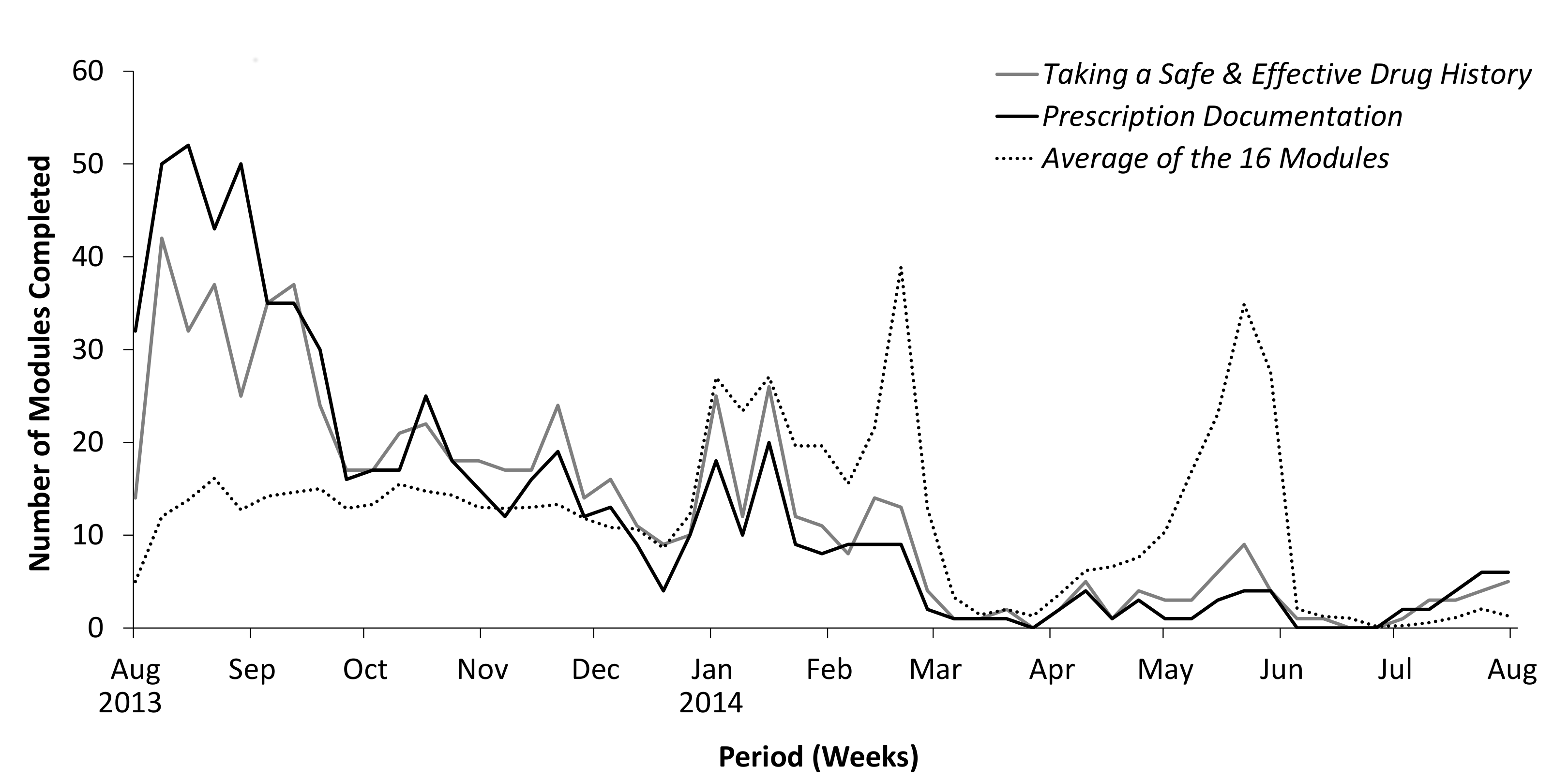

Supplement: Additional file 1: Figure S1. — Number of modules completed per week during Foundation Year 1 training (2013–14). (TIFF 376 kb) [file 12909_2016_651_MOESM1_ESM.tiff]
